# Supplementary material for: Single-cell immune repertoire atlas maps coordinated circulating adaptive immune states in inflammatory bowel disease
Source: bioRxiv. 2026 Sep 23:2026.09.20.753043. Preprint. [Version 1] doi: 10.64898/2026.09.20.753043 (PMC13622131; doi:10.64898/2026.09.20.753043)

bioRxiv preprint doi: https://doi.org/10.64898/2026.09.20.753043; this version posted September 23, 2026. The copyright holder has placed this preprint (which was not certified by peer review) in the Public Domain. It is no longer restricted by copyright. Anyone can legally share, reuse, remix, or adapt this material for any purpose without crediting the original authors.

Supplemental information

Supplemental figure legends

bioRxiv preprint doi: https://doi.org/10.64898/2026.09.20.753043; this version posted September 23, 2026. The copyright holder has placed this preprint (which was not certified by peer review) in the Public Domain. It is no longer restricted by copyright. Anyone can legally share, reuse, remix, or adapt this material for any purpose without crediting the original authors.

Figure S1. Receptor recovery and adaptive-state validation. (A) Productive paired alpha-beta, paired gamma-delta, and paired heavy-light receptor recovery across representative adaptive immune-cell states; dot size reports paired-cell number and color reports the percentage of cells within each state. (B) Canonical marker validation across representative CD4/Treg, CD8 T-cell, B-cell, and plasma-cell states; dot size indicates the percentage of cells expressing each gene and color indicates gene-wise scaled average expression. Both panels use the complete quality-controlled lineage datasets.

Figure S2. TCR repertoire structure and external motif context supporting Figures 2 and 3. (A) Participant-level diversity and clonality. (B) Diagnosis-stratified clone-size composition. (C) Cell-state composition of non-expanded and expanded exact paired alpha-beta clonotypes. (D) Carrier odds ratios for five previously reported GLIPH2 specificity motifs in the internal PBMC cohort and an independent European cohort. The external motif comparison supports the general existence of convergent IBD-associated motifs but does not validate the manuscript-specific sequence groups.

Figure S3. Independent longitudinal PBMC assessment of clone-associated programs, related to Figure 2. Public GSE261334 data [28] included five healthy participants and ten participants with UC sampled at baseline and week 6. (A) Baseline UC-minus-healthy program-score differences. (B) Paired week-6-minus-baseline changes in UC. Neither family contained FDR-significant effects. (C) Participant-level Spearman correlations between ordered exact paired-clone-size bins and program score for selected T- and B-cell programs; complementary TCR programs are shown in Figure 2H. (D) Baseline participant-level T–B program correlations ($n = 15$); no association passed FDR correction. Points in (A,B) show median effects without confidence intervals; daggers denote $0.05 \leq$ FDR <0.10. Boxes in (C) show participant medians and IQRs. Positive or negative clone-bin slopes are associations, not evidence that expansion selects or excludes entire cell lineages. Longitudinal changes were not tested as response effects because participant-linked response labels were unavailable for reanalysis.

Figure S4. BCR repertoire structure, expansion-associated programs, isotype, and SHM, related to Figures 4 and 5. (A) Participant-level diversity and clonality calculated directly from positive-count records in the IBDBCRonly source dataset. These row-based summaries differ from the CDR3-amino-acid-aggregated immunarch clonality shown in Figure 4A; their numerical values are not interchangeable. (B) Diagnosis-stratified clone-size composition. (C) State composition of expanded and nonexpanded paired-chain

bioRxiv preprint doi: https://doi.org/10.64898/2026.09.20.753043; this version posted September 23, 2026. The copyright holder has placed this preprint (which was not certified by peer review) in the Public Domain. It is no longer restricted by copyright. Anyone can legally share, reuse, remix, or adapt this material for any purpose without crediting the original authors.

BCR clonotypes. (D,E) CD- and UC-stratified legacy expanded-versus-nonexpanded program summaries, respectively; these are complementary to, not replacements for, the heavy-chain-defined state-matched pseudobulk contrasts in Figure 4C,D. (F) Class-switched fractions. (G) Diagnosis-level mean isotype composition and participant-level isotype fractions. (H) Heavy-chain SHM within matched B-cell states. Observed isotype fractions should be interpreted alongside the adjusted count models in Figure 4G. These analyses do not establish antigen specificity or an expansion-driven increase in plasma programming.

Figure S5. BCR germline-lineage reconstruction and definition sensitivity, related to Figure 5. (A) Participant-level regional heavy-chain SHM across framework and complementarity-determining regions. (B) Participant-level mean lineage minimum-spanning-tree branch length. (C) Sensitivity to normalized junction-distance thresholds of 0.10, 0.15, and 0.20. (D) Counts of heavy-chain lineages linked to both IgA-plasma and IgG-plasma transcriptional states: one detected lineage per diagnosis. These are detected lineage counts, not fractions, observed class-switch events, or estimates of trafficking. Light-chain-linked subdivisions and limited cell-to-full-heavy-sequence resolution constrain interpretation. The analyses distinguish accumulated germline distance from diversification among observed descendants.

Figure S6. BCR lineage maturation and clinical context, related to Figure 5. (A) Participant-aware associations of five state-residualized, constant-region-excluded programs with germline distance, branch length among diversified lineages, class-switching probability, and cross-state occupancy. Continuous outcomes are standardized; switching effects are absolute probabilities. Models adjusted for lineage abundance, scored-cell depth, and pseudotime, with participant fixed effects and participant-clustered errors; FDR was controlled across 20 tests. (B) Objective-inflammation contrasts. (C) Associations with continuous fecal calprotectin. (D) Mucosal-state composition of expanded lineages. (E) Paired-light-chain context. Points and intervals denote the corresponding participant-aware estimates and 95% CIs where shown. In (B–E), blue and orange denote CD and UC, respectively; filled symbols indicate FDR <0.05. These cross-sectional analyses do not establish mutation rate, temporal switch direction, antigen specificity, or an effect of exact-clonotype expansion on plasma programming.

Figure S7. Global TCR–BCR coordination and robustness, related to Figure 6. (A) Complete participant-level cross-compartment partial-correlation screen. (B–D) Representative repertoire associations; scatterplots show unadjusted participant values,

bioRxiv preprint doi: https://doi.org/10.64898/2026.09.20.753043; this version posted September 23, 2026. The copyright holder has placed this preprint (which was not certified by peer review) in the Public Domain. It is no longer restricted by copyright. Anyone can legally share, reuse, remix, or adapt this material for any purpose without crediting the original authors.

whereas annotations report covariate-adjusted partial correlations. (E) Diagnosis-stratified cytotoxic–plasma program associations. (F) In-sample model variance and incremental information; these R^2 summaries differ from the held-out correlations in Figure 6F. (G) Leave-one-acquisition-series influence. (H) Cell-composition sensitivity. (I) Contemporaneous clinical associations. (J) Cross-program specificity in CD. (K) Formal between-program contrasts, none significant. In (A,J), asterisks indicate FDR <0.05 (*) or <0.01 (**). Covariates and resampling are specified in STAR Methods. The analyses assess covariation, not direct interaction or mediation.

Figure S8. Helper, Th17, and Treg clone architecture and B-cell coordination, related to Figure 6. (A) Exact paired αβ Th17/Treg clone composition. (B) Within-participant state-label null for mixed clones (10,000 permutations); eight observed mixed IBD clones versus 14.57 expected on average. Gray intervals show the central 95% of permutation-null counts; diamonds mark observed counts. (C) Clone-size effects. (D) State-matched expanded-minus-singleton program contrasts. (E) Participant-level helper/regulatory–B-cell partial correlations; asterisks indicate FDR <0.05 (*), <0.01 (**), or <0.001 (***). (F) Independent standardized joint-model contributions. (G) Representative adjusted pathogenic-Th17–plasma-differentiation association. (H) Leave-one-acquisition-series sensitivity. (I) CD-versus-UC interactions restricted to shared series; none passed FDR correction. Clone-size and expansion contrasts for the focused Th17/Treg programs were not FDR significant. Mixed-clone depletion distinguishes clone sharing from participant-level coordination but does not establish the mechanism of that coordination.

Figure S9. External mucosal and blood–tissue analyses, related to Figures 2, 5, and 6. (A) Rectal Th17-like and Treg expanded-cell fractions in GSE125527 [17]. (B) Exact paired TCR or BCR clonotypes shared with blood. (C) BCR clone-bin/program associations in the same external resource. Disease comparisons did not pass FDR correction. (D) Program-score differences between blood-shared and colon-private TCR clonotypes in three CD participants from GSE301689 [32]. These n = 3 estimates are descriptive, and none passed FDR correction. Cross-tissue identity does not establish trafficking direction; the public BCR files lacked the germline-alignment information needed to validate the SHM findings.

Figure S10. Diagnosis benchmarking, calibration, and selection optimism, related to Figure 7. (A) Nested participant-level performance of fixed primary diagnosis models and available comparator representations. (B) Observed performance and 25 fixed-

bioRxiv preprint doi: https://doi.org/10.64898/2026.09.20.753043; this version posted September 23, 2026. The copyright holder has placed this preprint (which was not certified by peer review) in the Public Domain. It is no longer restricted by copyright. Anyone can legally share, reuse, remix, or adapt this material for any purpose without crediting the original authors.

pipeline label-permutation results; these permutations do not repeat the complete nested tuning or historical model search. (C) Representative CD-versus-control calibration curves; threshold-dependent metrics are provided in Source Data. (D) Historical best-of-screen AUC minus pooled nested out-of-fold AUC for the primary model. Outer validation used five stratified folds repeated three times and inner three-fold tuning. Fold-median intervals bootstrap 15 correlated outer-fold summaries and are not independent-cohort CIs. Comparator analyses may use different eligible participants; no matched head-to-head superiority is inferred. No external clinical-classifier transfer is included.

Supplemental table titles

Table S1. Clinical and demographic characteristics of the study cohort. Participant-level demographic and clinical characteristics for the PBMC2026 cohort, reported overall and by non-IBD control, ulcerative colitis, and Crohn's disease groups. Continuous variables are summarized by median, interquartile range, and range where available; categorical variables are reported as number and percentage.

Table S2. Milo differential-abundance results for CD versus non-IBD control PBMC neighborhoods. Full Milo differential-abundance output for the CD versus control comparison across 62,426 PBMC neighborhoods. Columns report log fold change (logFC), average log counts per million (logCPM), F statistic, nominal P value, Benjamini-Hochberg FDR, neighborhood identifier (Nhood), spatial false-discovery-rate-adjusted value (SpatialFDR), neighborhood center cell identifier, assigned immune-cell type, and the fraction of cells in the neighborhood assigned to that cell type. Positive logFC values indicate enrichment in CD relative to control; negative values indicate enrichment in controls.

Table S3. Milo differential-abundance results for UC versus non-IBD control PBMC neighborhoods. Full Milo differential-abundance output for the UC versus control comparison across 38,371 PBMC neighborhoods. Columns report logFC, logCPM, F statistic, nominal P value, FDR, Nhood, SpatialFDR, neighborhood center cell identifier, assigned immune-cell type, and cell-type fraction. Positive logFC values indicate enrichment in UC relative to control; negative values indicate enrichment in controls.

Table S4. Milo differential-abundance results for CD versus UC PBMC neighborhoods. Full Milo differential-abundance output for the CD versus UC comparison across 66,508 PBMC neighborhoods. Columns report logFC, logCPM, F statistic, nominal P value, FDR, Nhood, SpatialFDR, neighborhood center cell identifier, assigned immune-cell type, and cell-type fraction. Positive logFC values indicate enrichment in CD relative to UC; negative values indicate enrichment in UC.

Table S5. TCR repertoire model-screen performance across diagnosis, inflammation, and contemporaneous response-status tasks. The workbook contains 594 model–comparison records and separate summaries for ROC AUC, balanced

bioRxiv preprint doi: https://doi.org/10.64898/2026.09.20.753043; this version posted September 23, 2026. The copyright holder has placed this preprint (which was not certified by peer review) in the Public Domain. It is no longer restricted by copyright. Anyone can legally share, reuse, remix, or adapt this material for any purpose without crediting the original authors.

accuracy, Matthews correlation coefficient, and F1 score. It documents the broader historical screen, including available linear, DeepRC, and diversity/clonality models, rather than replacing the primary nested estimates in Figure 7B or conditional out-of-fold estimates in Figure 7D,F. Evaluation design and eligibility must be read with each record.

Table S6. BCR repertoire model-screen performance across diagnosis, inflammation, and contemporaneous response-status tasks. The workbook contains 522 model–comparison records, with task-specific ROC AUC, balanced accuracy, Matthews correlation coefficient, and F1 summaries. It documents historical candidate models and representations, not a uniform nested evaluation of every candidate. The primary diagnosis and conditional clinical-state estimates are reported in Figure 7 and its Source Data; individual records retain their own feature definitions, eligible samples, and evaluation designs.

Table S7. Core TCR and BCR transcriptional gene signatures. The workbook contains 17 T-cell and 19 B-cell modules, their identifiers, labels, ordered gene sets, and long-format module–gene associations. Scores use the mean normalized expression of detected signature genes. Focused pathogenic/conventional Th17 and suppressive/reprogramming Treg definitions added in subsequent analyses are specified separately in STAR Methods and the analysis code; Table S7 is not an exhaustive list of every focused analysis-specific variant.

bioRxiv preprint doi: https://doi.org/10.64898/2026.09.20.753043; this version posted September 23, 2026. The copyright holder has placed this preprint (which was not certified by peer review) in the Public Domain. It is no longer restricted by copyright. Anyone can legally share, reuse, remix, or adapt this material for any purpose without crediting the original authors.

Figure S1. Receptor recovery and adaptive-state validation

A **Paired-receptor recovery across representative adaptive immune-cell states**

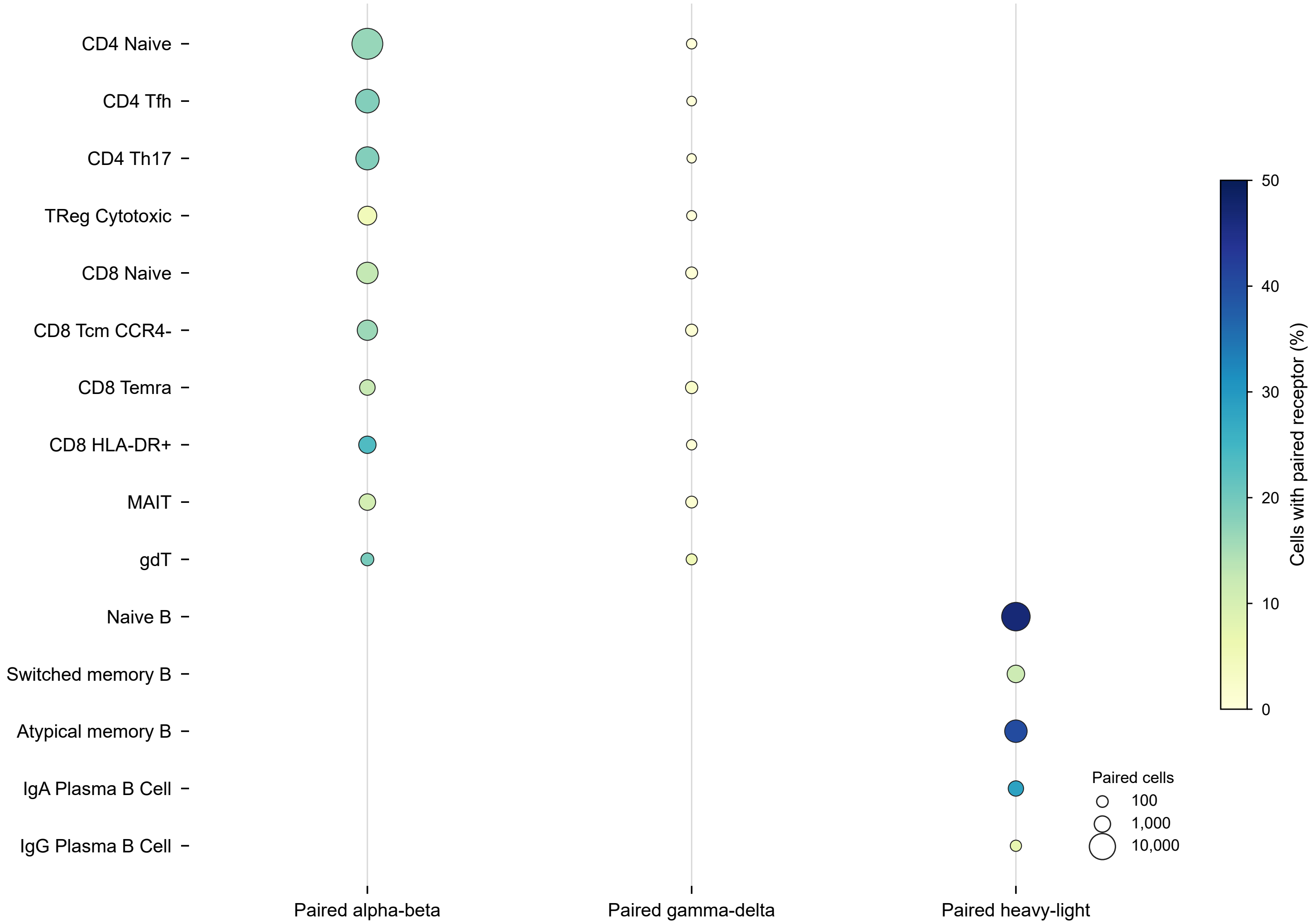

bioRxiv preprint doi: https://doi.org/10.64898/2026.09.20.753043; this version posted September 23, 2026. The copyright holder has placed this preprint (which was not certified by peer review) in the Public Domain. It is no longer restricted by copyright. Anyone can legally share, reuse, remix, or adapt this material for any purpose without crediting the original authors.

B Canonical marker validation of adaptive immune-cell states

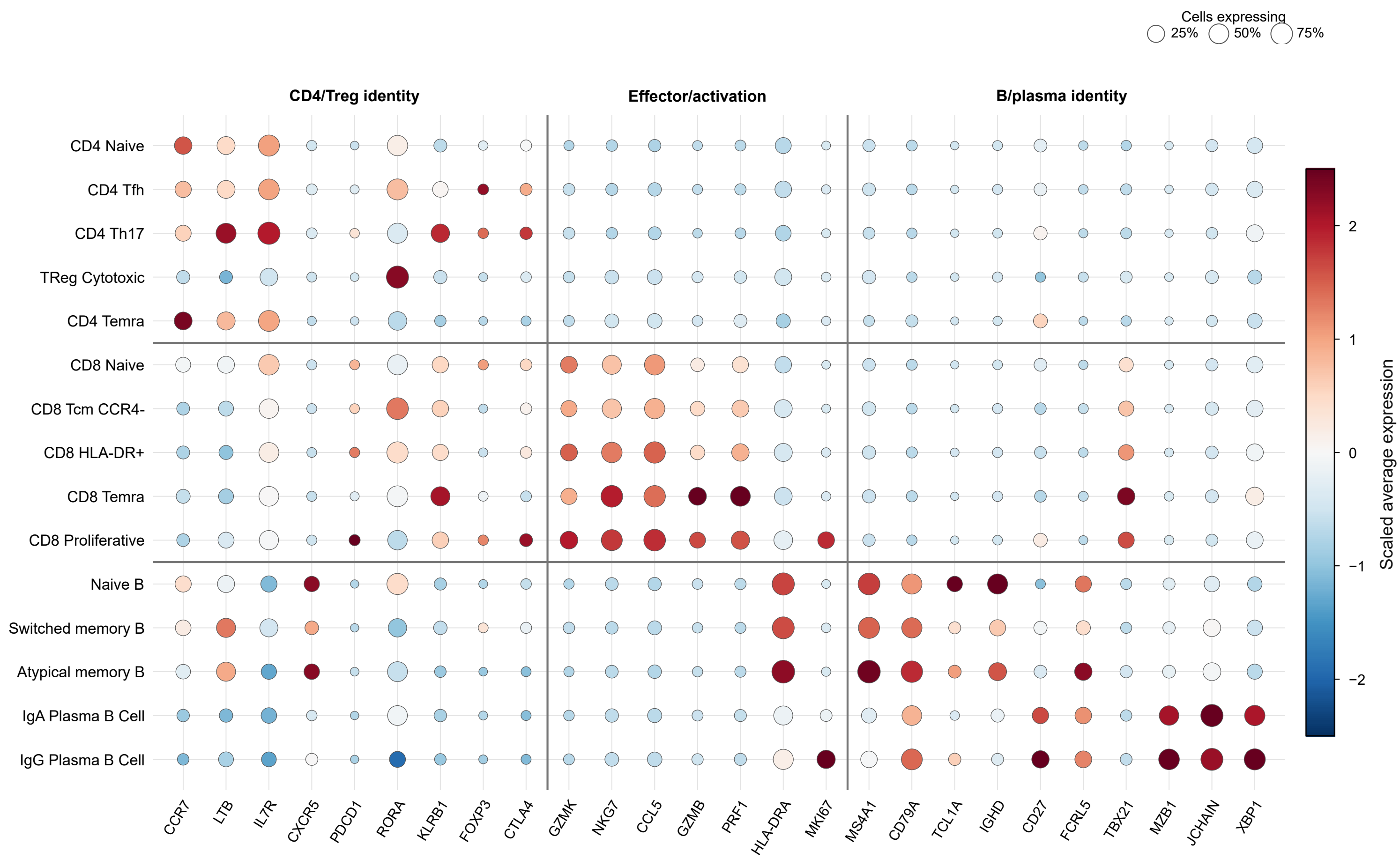

bioRxiv preprint doi: https://doi.org/10.64898/2026.09.20.753043; this version posted September 23, 2026. The copyright holder has placed this preprint (which was not certified by peer review) in the Public Domain. It is no longer restricted by copyright. Anyone can legally share, reuse, remix, or adapt this material for any purpose without crediting the original authors.

Figure S2. TCR repertoire structure and external motif context

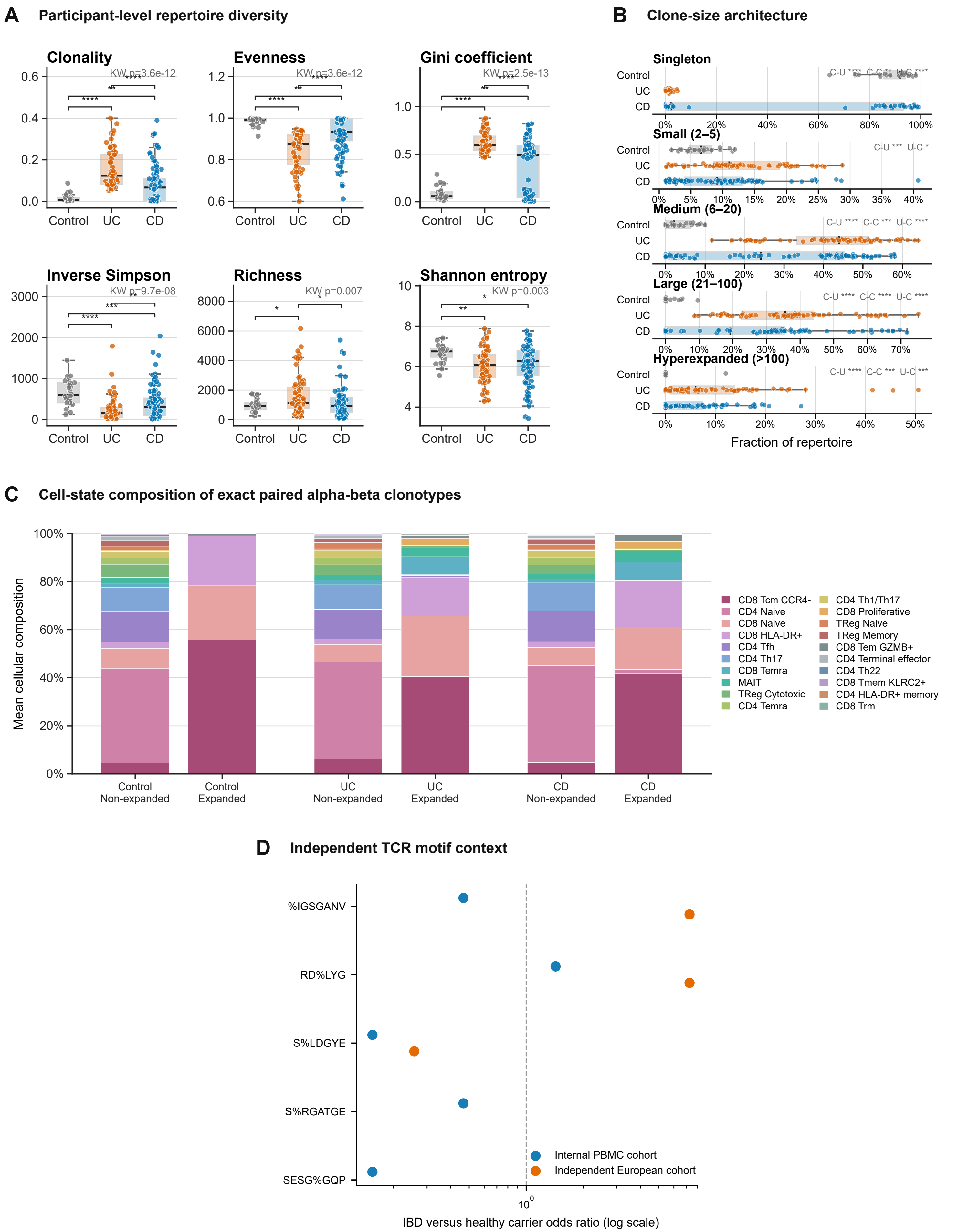

bioRxiv preprint doi: https://doi.org/10.64898/2026.09.20.753043; this version posted September 23, 2026. The copyright holder has placed this preprint (which was not certified by peer review) in the Public Domain. It is no longer restricted by copyright. Anyone can legally share, reuse, remix, or adapt this material for any purpose without crediting the original authors.

Figure S3. Independent longitudinal PBMC validation

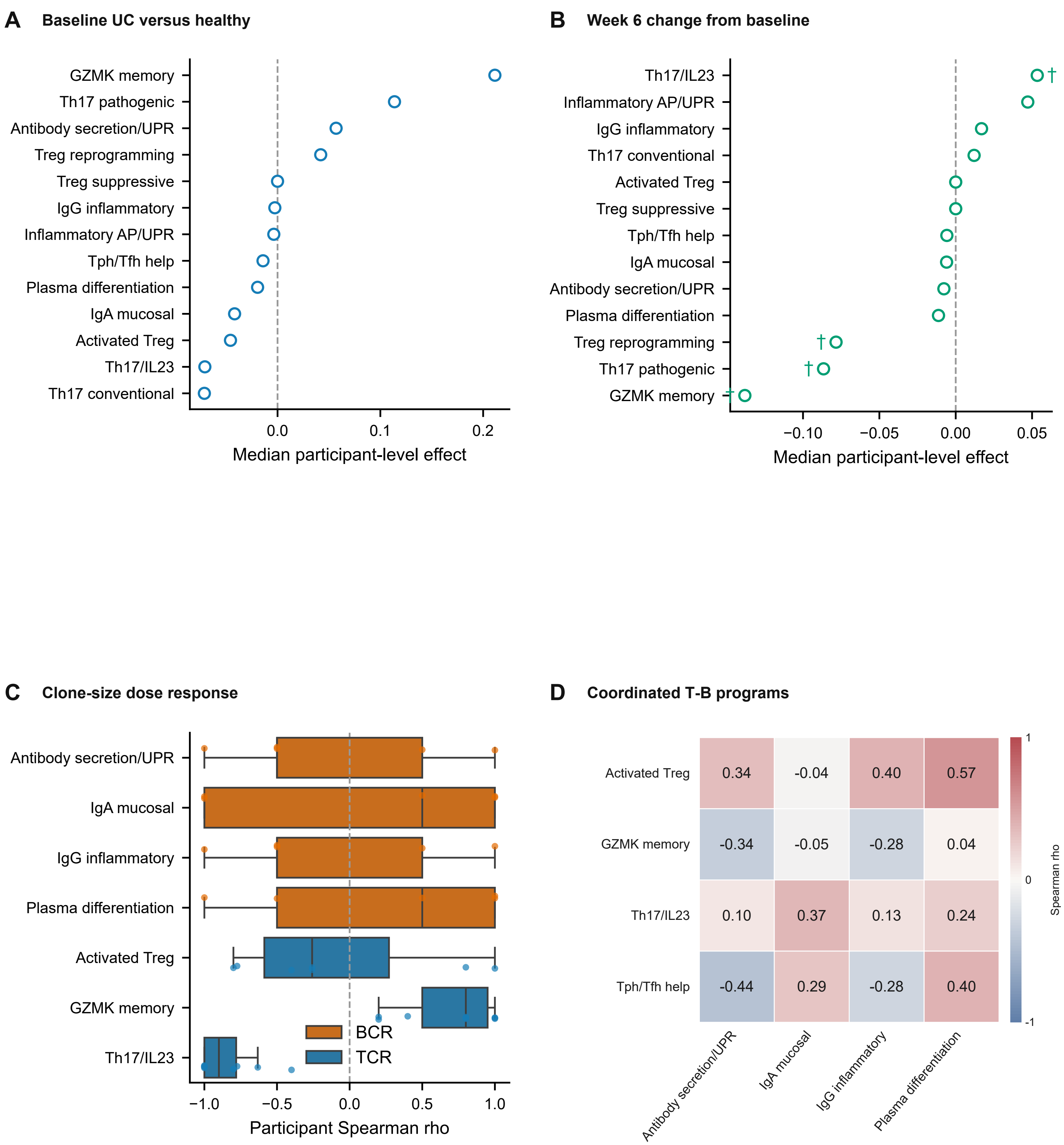

bioRxiv preprint doi: https://doi.org/10.64898/2026.09.20.753043; this version posted September 23, 2026. The copyright holder has placed this preprint (which was not certified by peer review) in the Public Domain. It is no longer restricted by copyright. Anyone can legally share, reuse, remix, or adapt this material for any purpose without crediting the original authors.

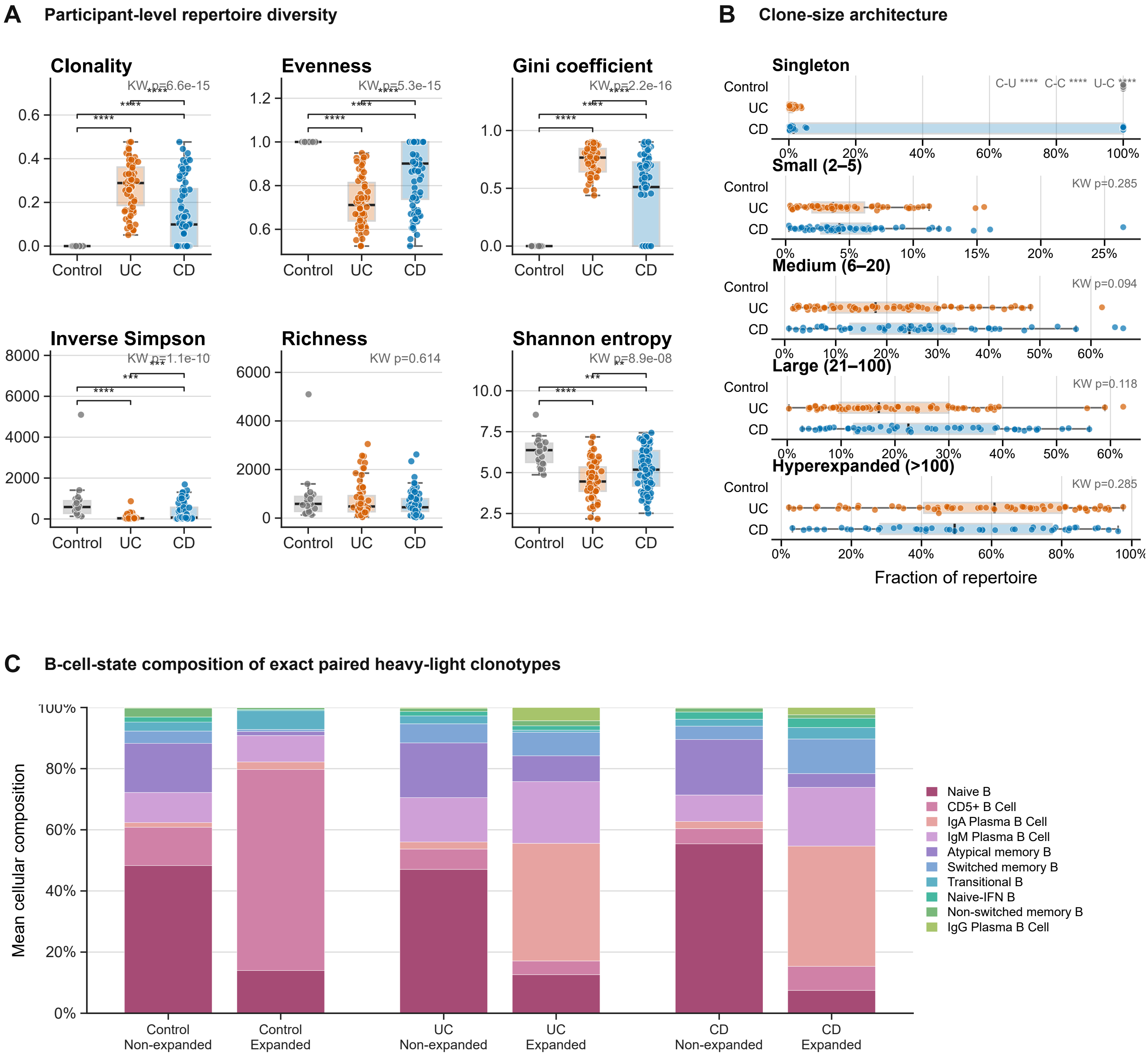

bioRxiv preprint doi: https://doi.org/10.64898/2026.09.20.753043; this version posted September 23, 2026. The copyright holder has placed this preprint (which was not certified by peer review) in the Public Domain. It is no longer restricted by copyright. Anyone can legally share, reuse, remix, or adapt this material for any purpose without crediting the original authors.

D Crohn's disease: expansion-linked B-cell programs

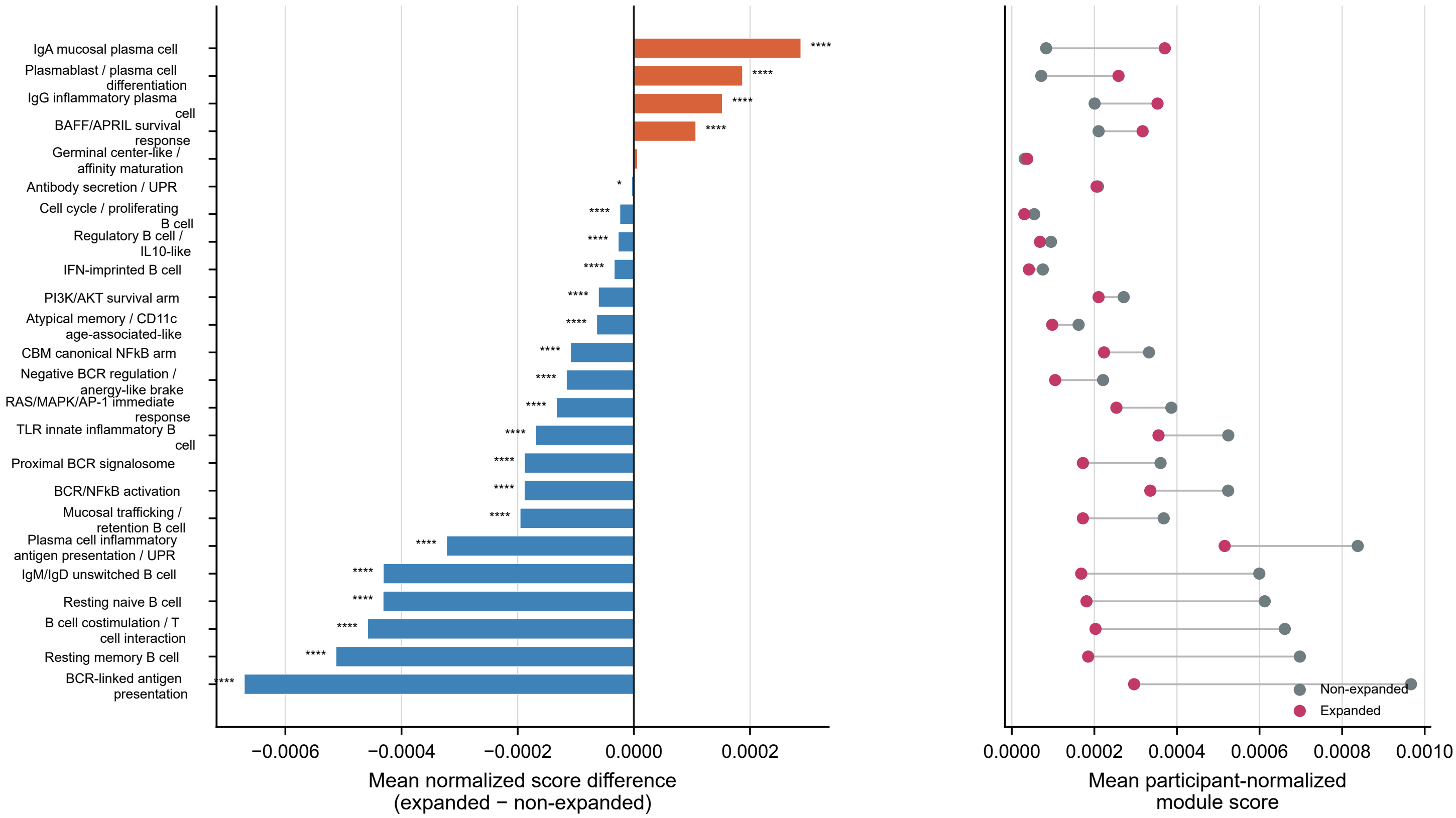

E Ulcerative colitis: expansion-linked B-cell programs

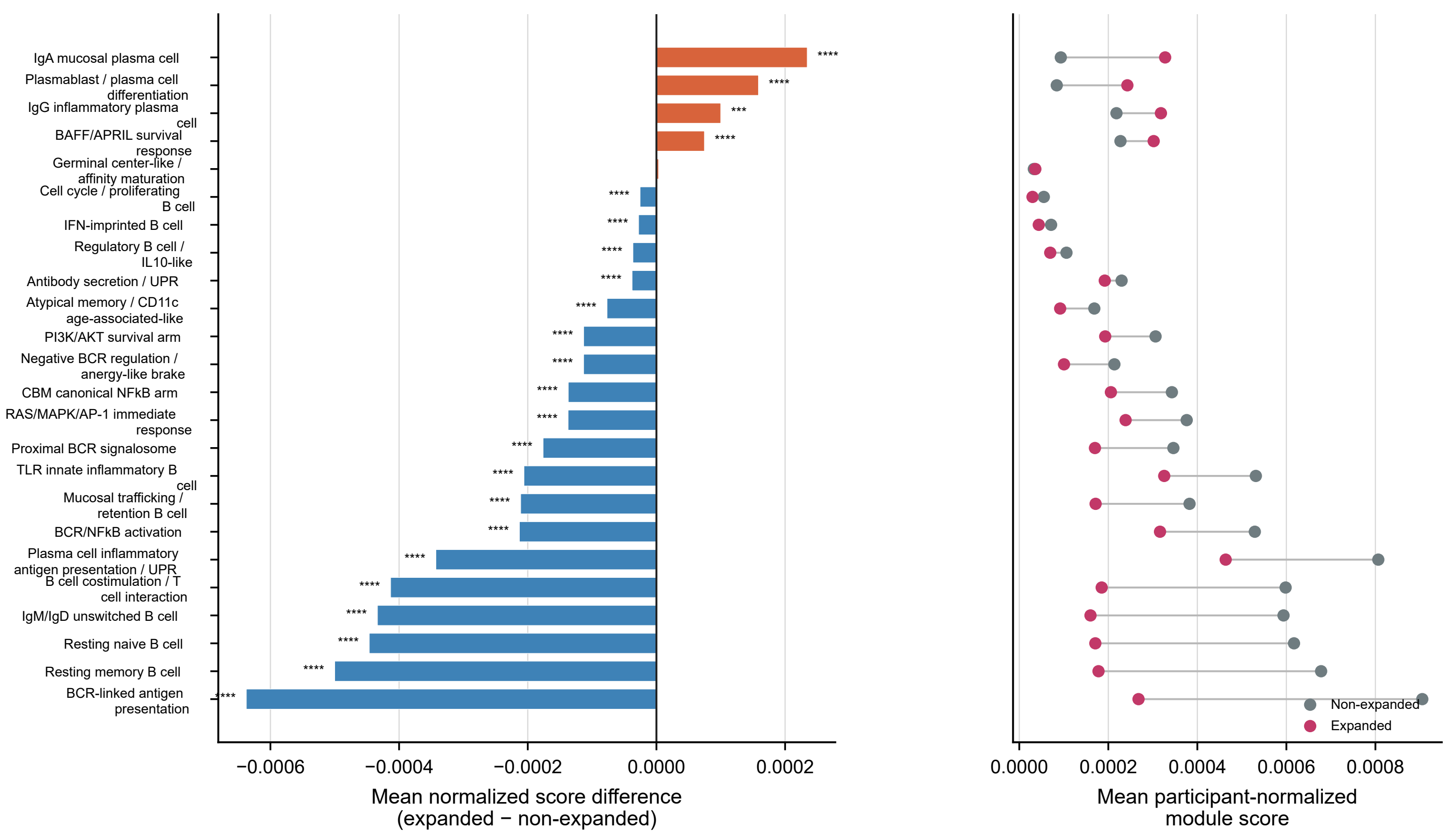

bioRxiv preprint doi: https://doi.org/10.64898/2026.09.20.753043; this version posted September 23, 2026. The copyright holder has placed this preprint (which was not certified by peer review) in the Public Domain. It is no longer restricted by copyright. Anyone can legally share, reuse, remix, or adapt this material for any purpose without crediting the original authors.

Figure S4. BCR repertoire structure, programs, and maturation

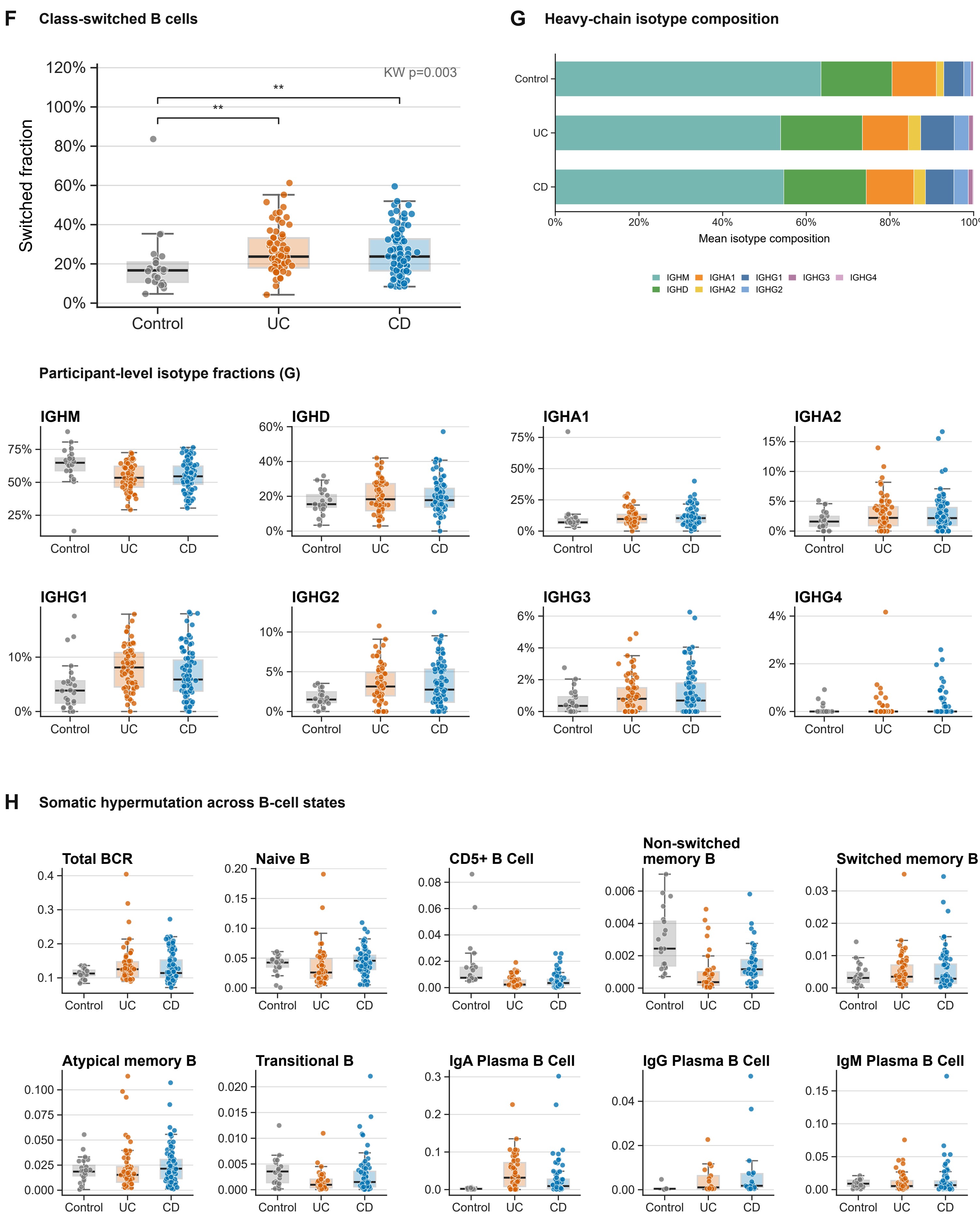

Boxes show median and interquartile range; points denote participants. *FDR < 0.05; **FDR < 0.01; ***FDR < 0.001; ****FDR < 0.0001.

bioRxiv preprint doi: https://doi.org/10.64898/2026.09.20.753043; this version posted September 23, 2026. The copyright holder has placed this preprint (which was not certified by peer review) in the Public Domain. It is no longer restricted by copyright. Anyone can legally share, reuse, remix, or adapt this material for any purpose without crediting the original authors.

Figure S5. BCR germline-lineage definition sensitivity

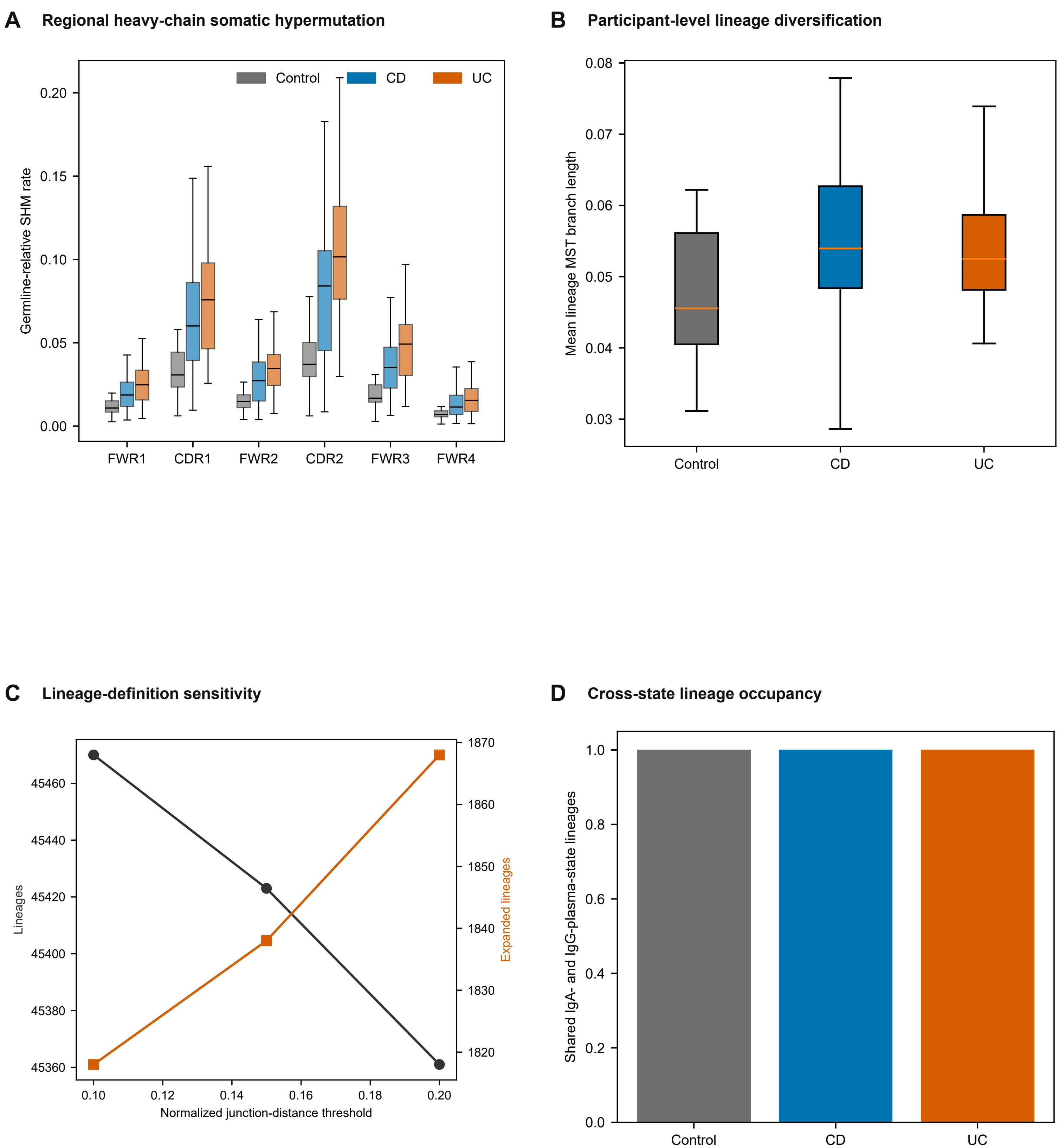

bioRxiv preprint doi: https://doi.org/10.64898/2026.09.20.753043; this version posted September 23, 2026. The copyright holder has placed this preprint (which was not certified by peer review) in the Public Domain. It is no longer restricted by copyright. Anyone can legally share, reuse, remix, or adapt this material for any purpose without crediting the original authors.

A **State-residualized B-cell programs and lineage maturation**

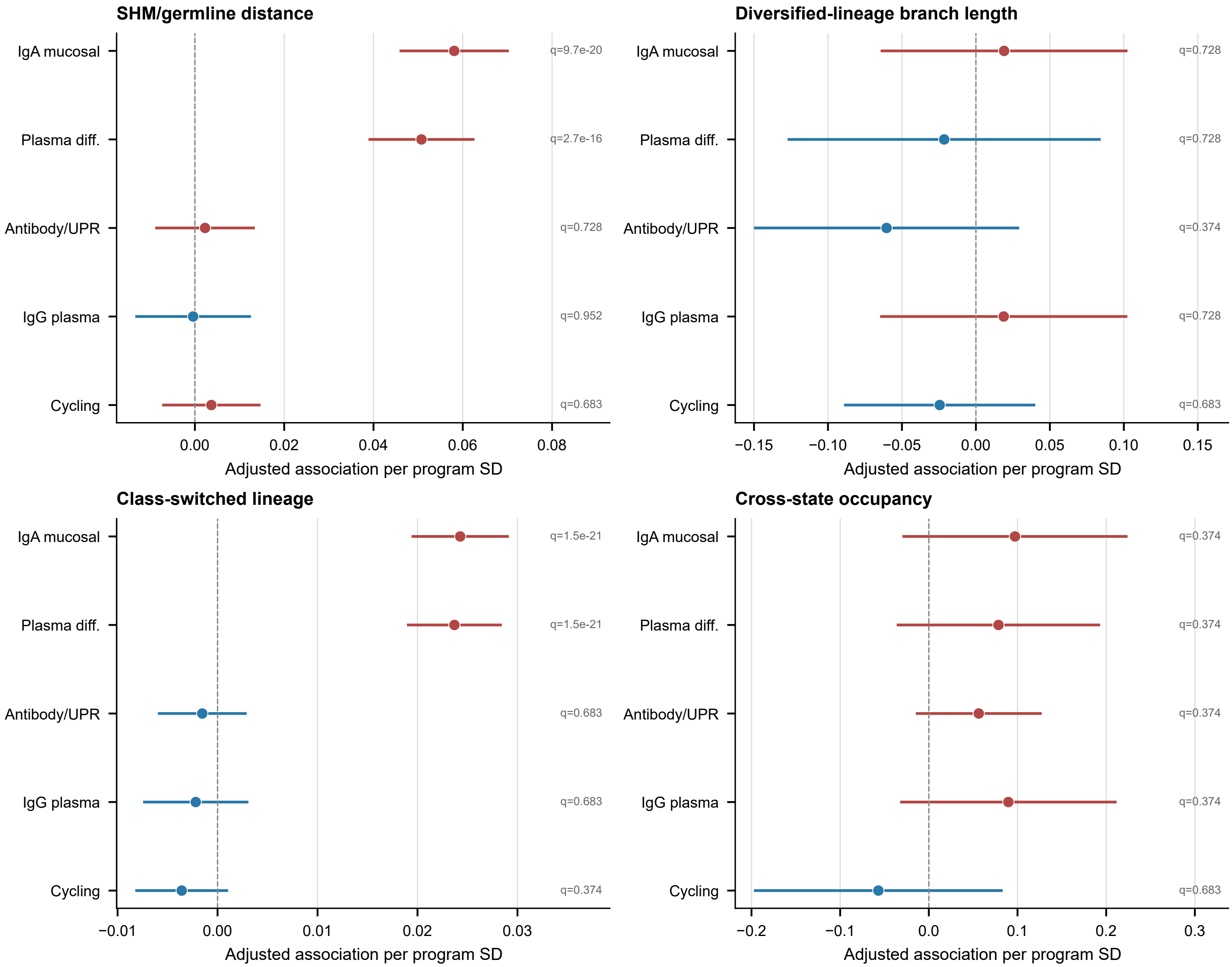

bioRxiv preprint doi: https://doi.org/10.64898/2026.09.20.753043; this version posted September 23, 2026. The copyright holder has placed this preprint (which was not certified by peer review) in the Public Domain. It is no longer restricted by copyright. Anyone can legally share, reuse, remix, or adapt this material for any purpose without crediting the original authors.

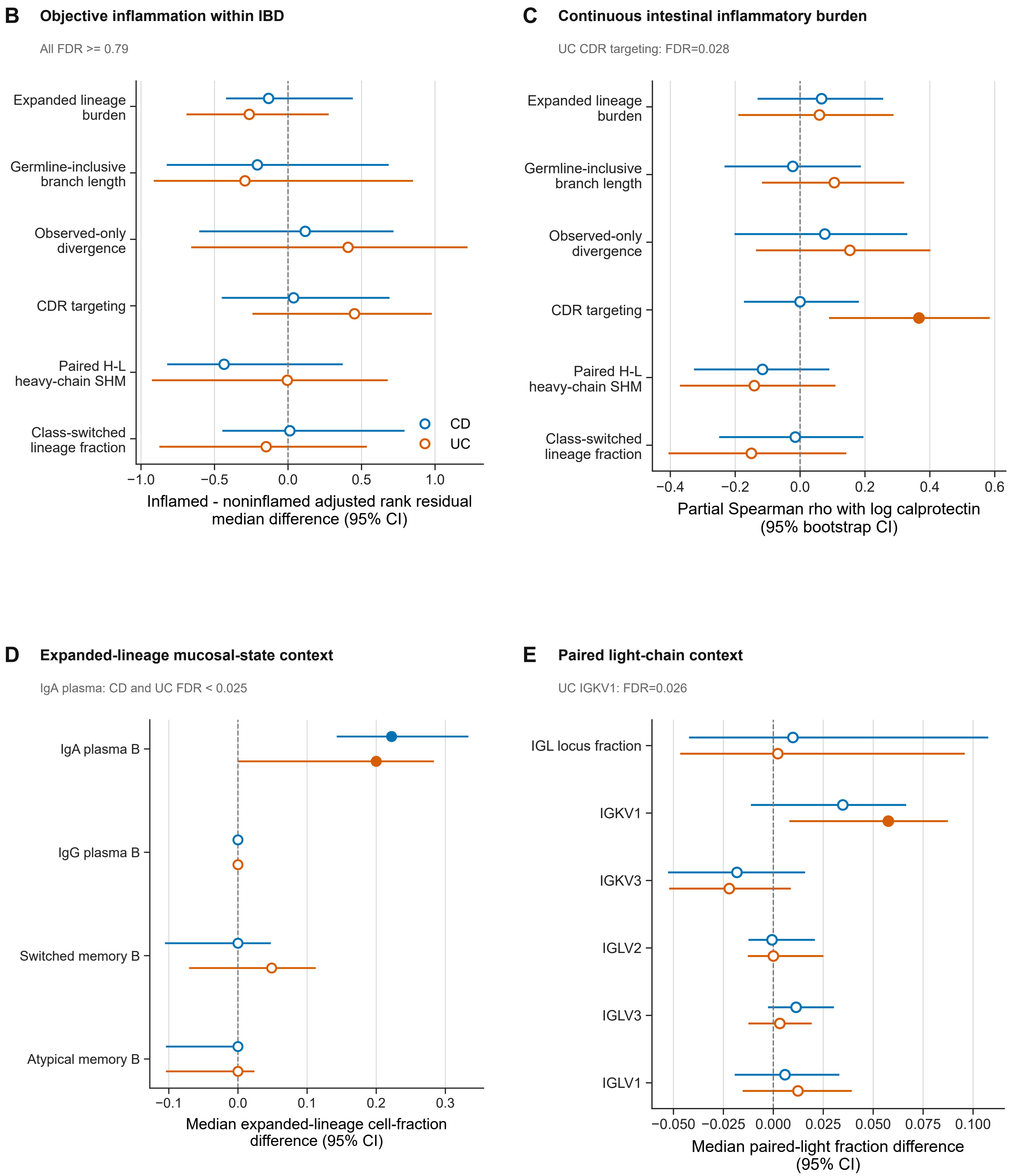

bioRxiv preprint doi: https://doi.org/10.64898/2026.09.20.753043; this version posted September 23, 2026. The copyright holder has placed this preprint (which was not certified by peer review) in the Public Domain. It is no longer restricted by copyright. Anyone can legally share, reuse, remix, or adapt this material for any purpose without crediting the original authors.

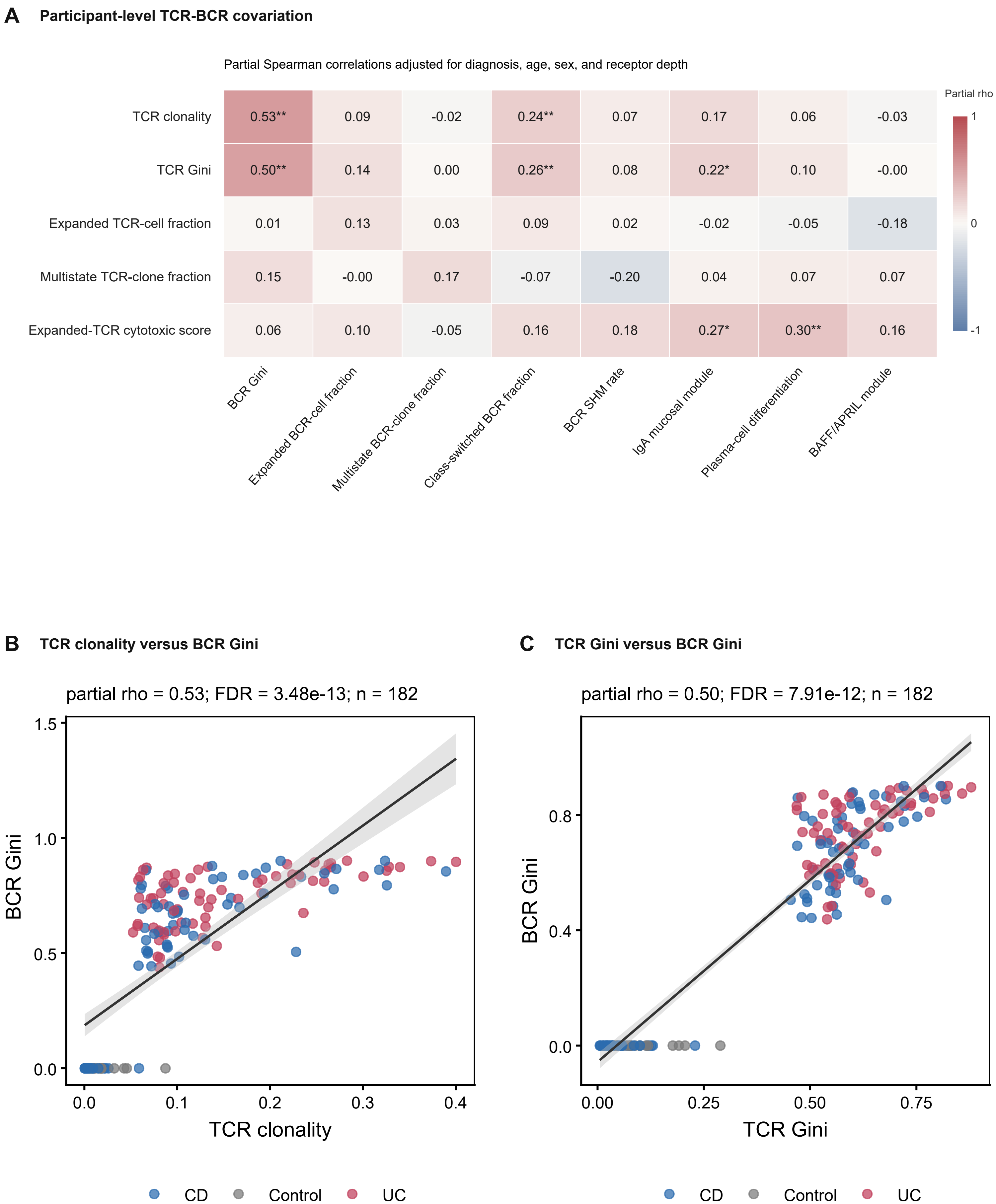

bioRxiv preprint doi: https://doi.org/10.64898/2026.09.20.753043; this version posted September 23, 2026. The copyright holder has placed this preprint (which was not certified by peer review) in the Public Domain. It is no longer restricted by copyright. Anyone can legally share, reuse, remix, or adapt this material for any purpose without crediting the original authors.

D **TCR Gini versus class-switched BCR fraction**

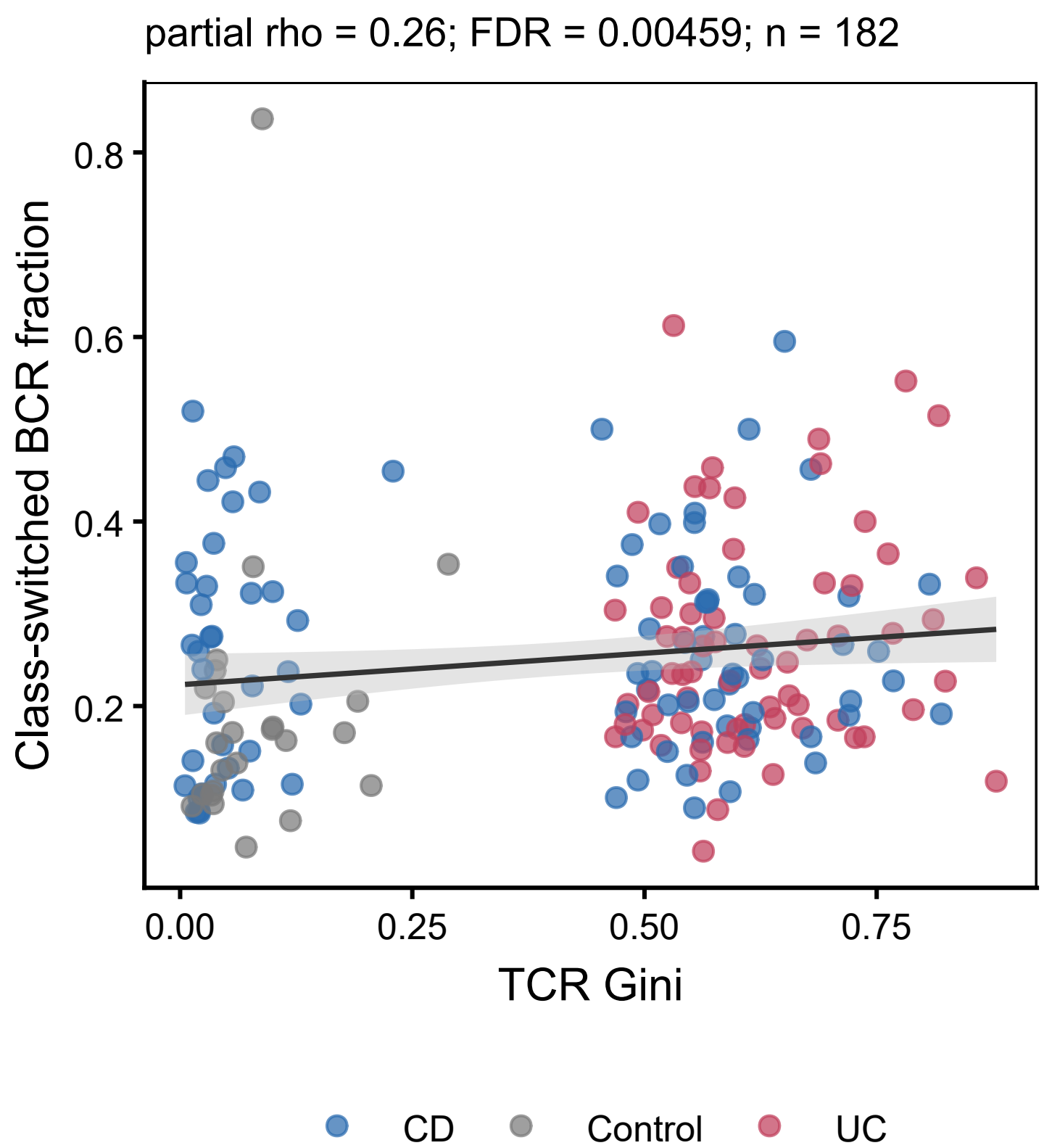

E **Cytotoxic T-cell and plasma-cell programs covary selectively in Crohn's disease**

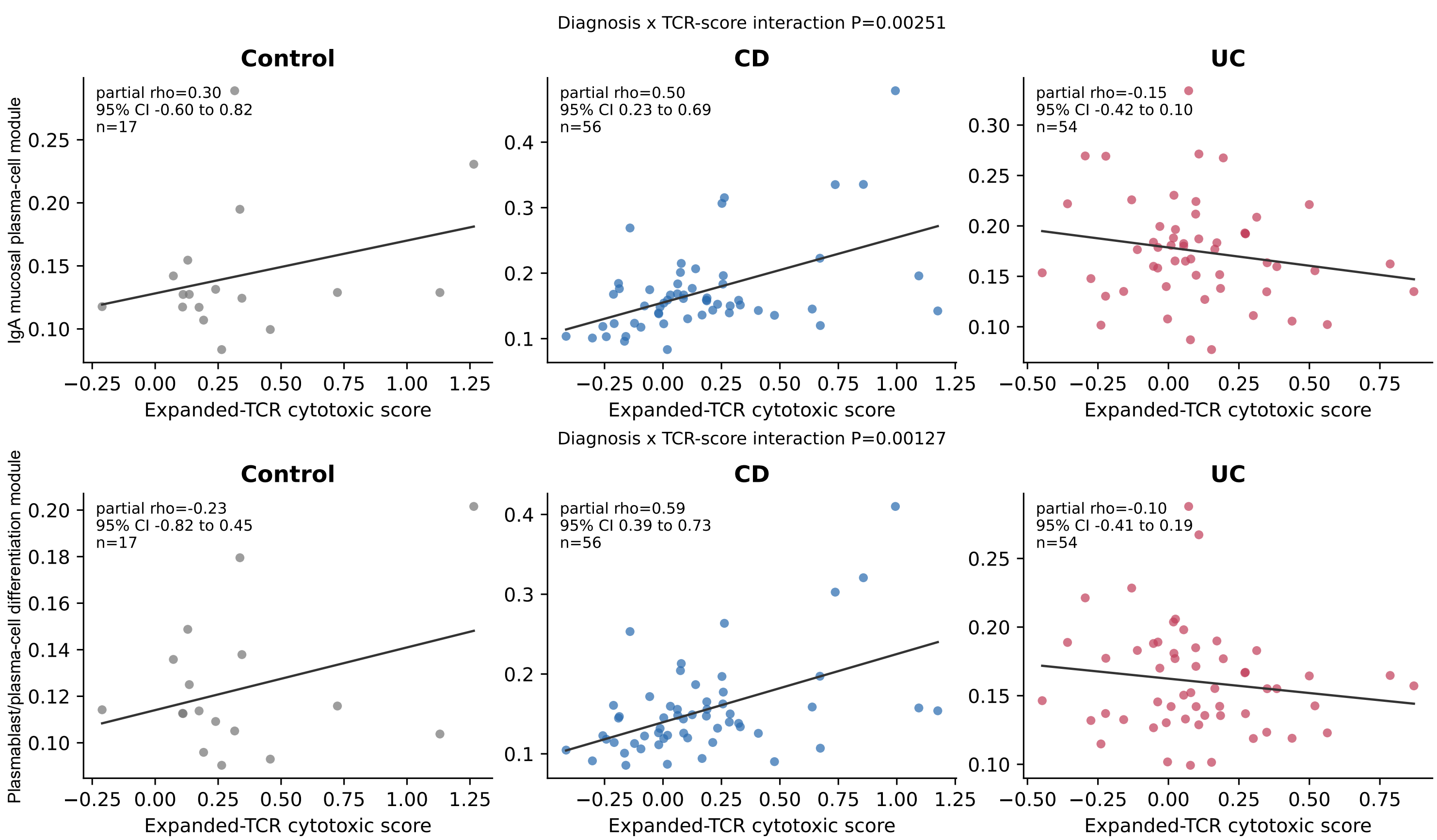

bioRxiv preprint doi: https://doi.org/10.64898/2026.09.20.753043; this version posted September 23, 2026. The copyright holder has placed this preprint (which was not certified by peer review) in the Public Domain. It is no longer restricted by copyright. Anyone can legally share, reuse, remix, or adapt this material for any purpose without crediting the original authors.

Figure S7. Global TCR-BCR coordination and robustness

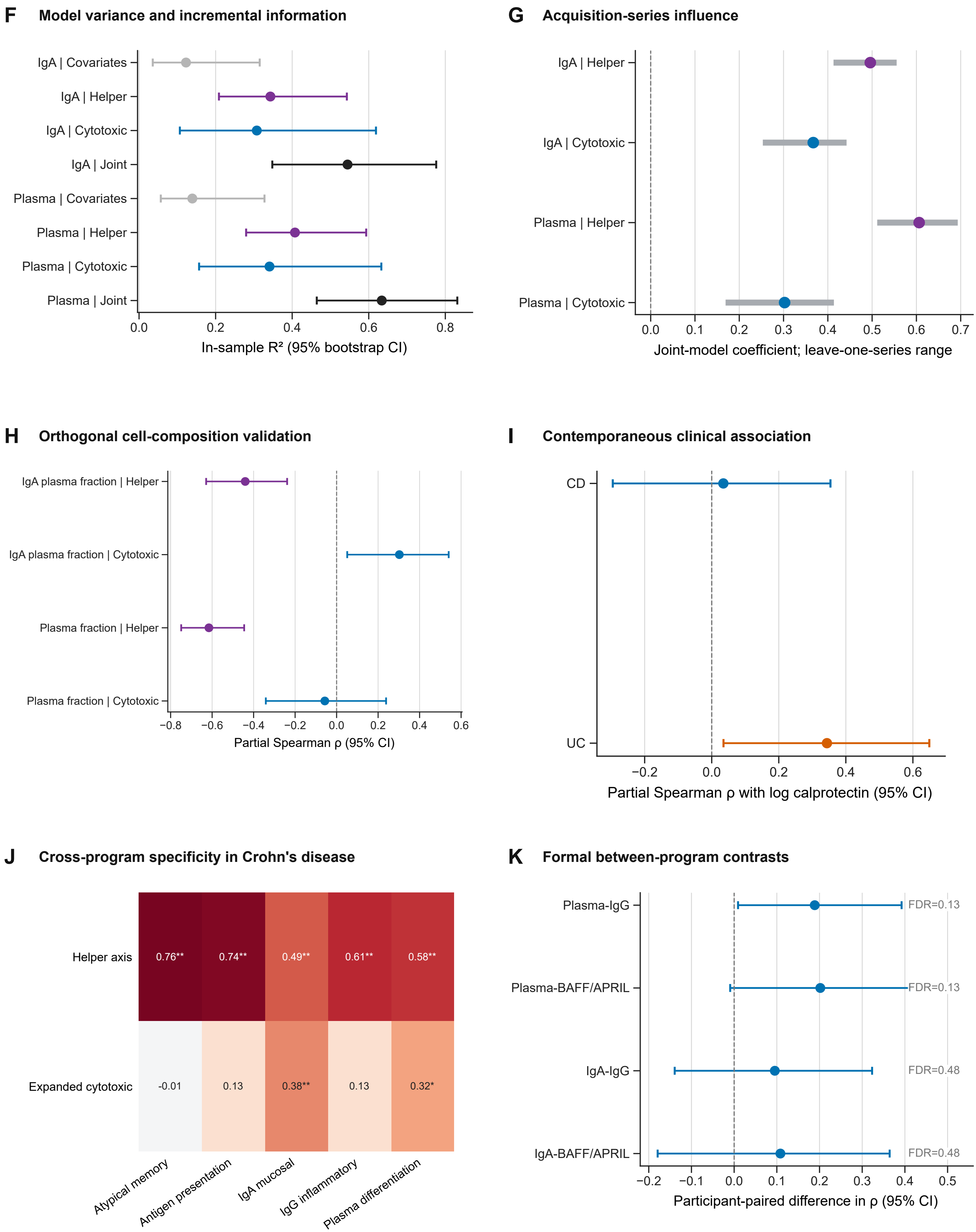

bioRxiv preprint doi: https://doi.org/10.64898/2026.09.20.753043; this version posted September 23, 2026. The copyright holder has placed this preprint (which was not certified by peer review) in the Public Domain. It is no longer restricted by copyright. Anyone can legally share, reuse, remix, or adapt this material for any purpose without crediting the original authors.

Figure S8. Helper, Th17, and Treg analyses

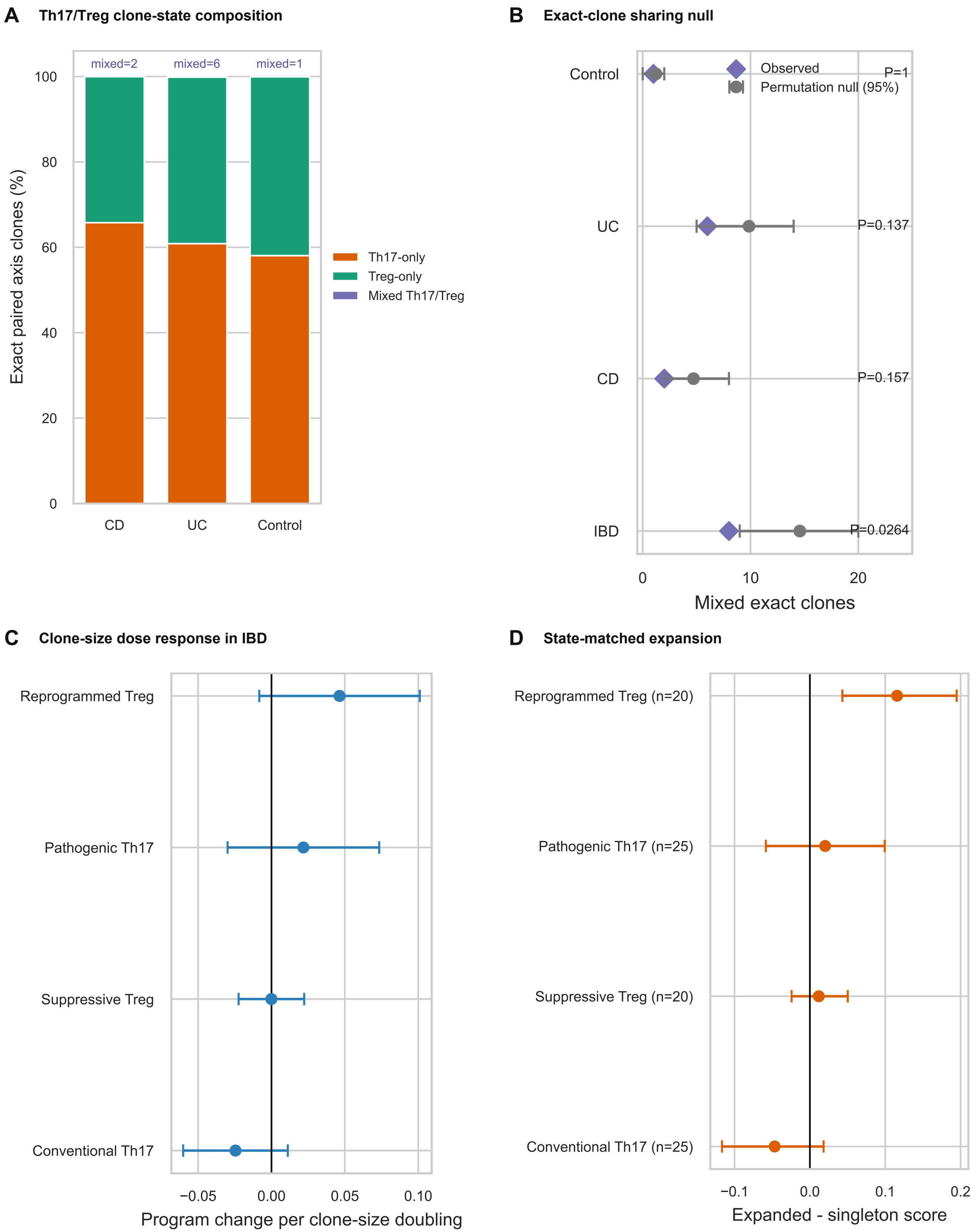

bioRxiv preprint doi: https://doi.org/10.64898/2026.09.20.753043; this version posted September 23, 2026. The copyright holder has placed this preprint (which was not certified by peer review) in the Public Domain. It is no longer restricted by copyright. Anyone can legally share, reuse, remix, or adapt this material for any purpose without crediting the original authors.

E **Targeted T-cell-B-cell coupling in IBD**

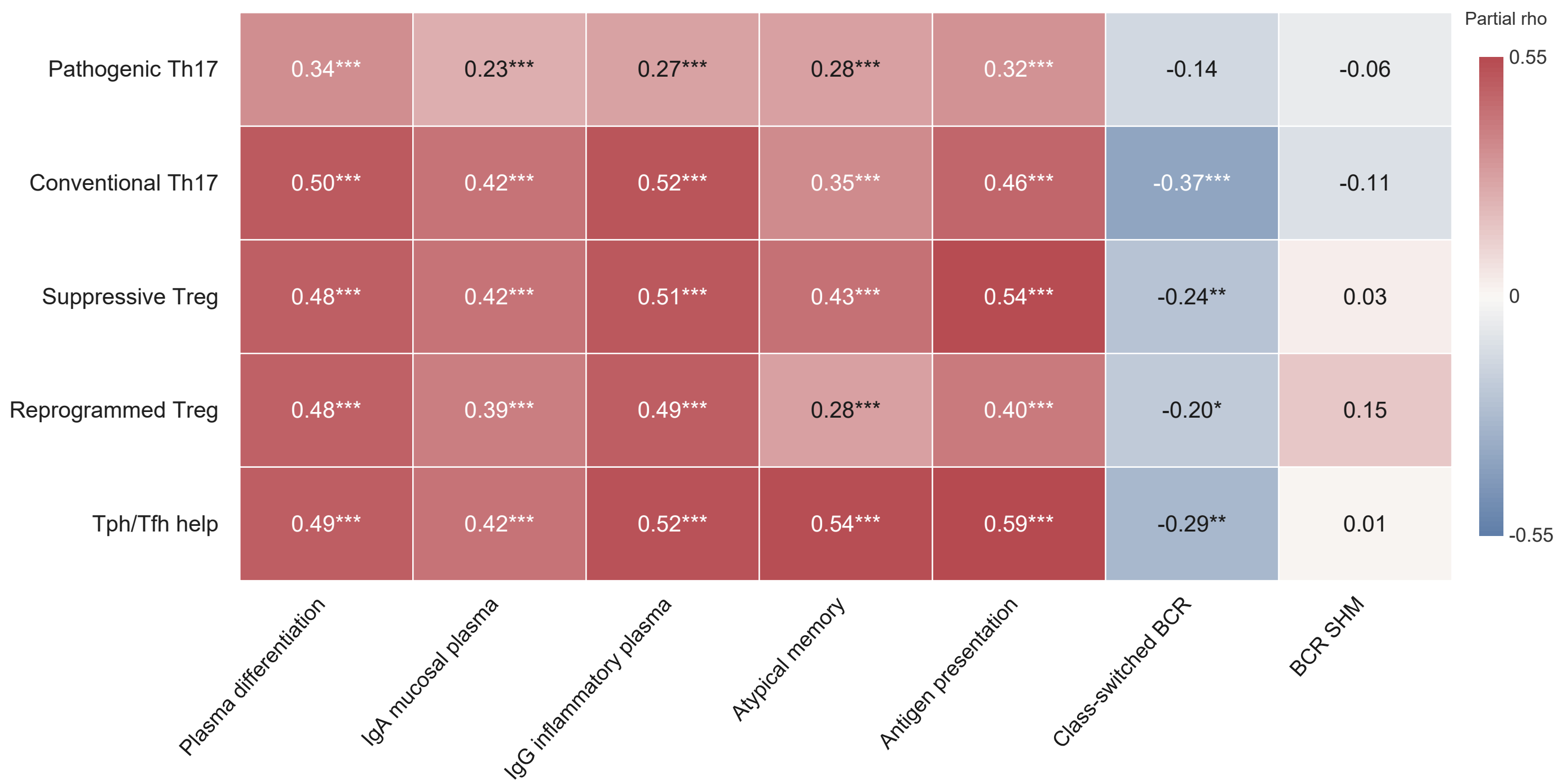

F **Independent helper model**

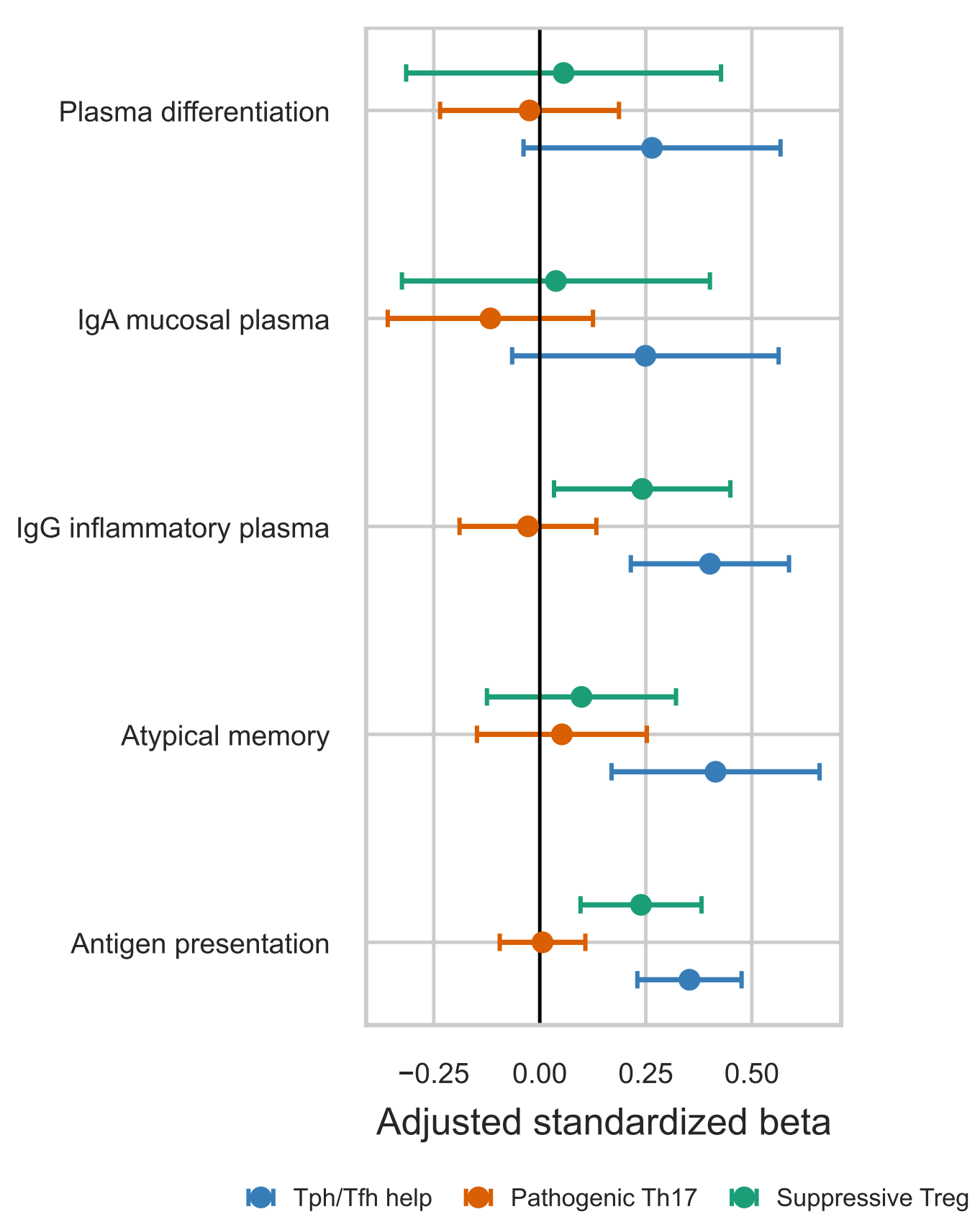

G **Representative adjusted association: Pathogenic Th17 versus plasma differentiation**

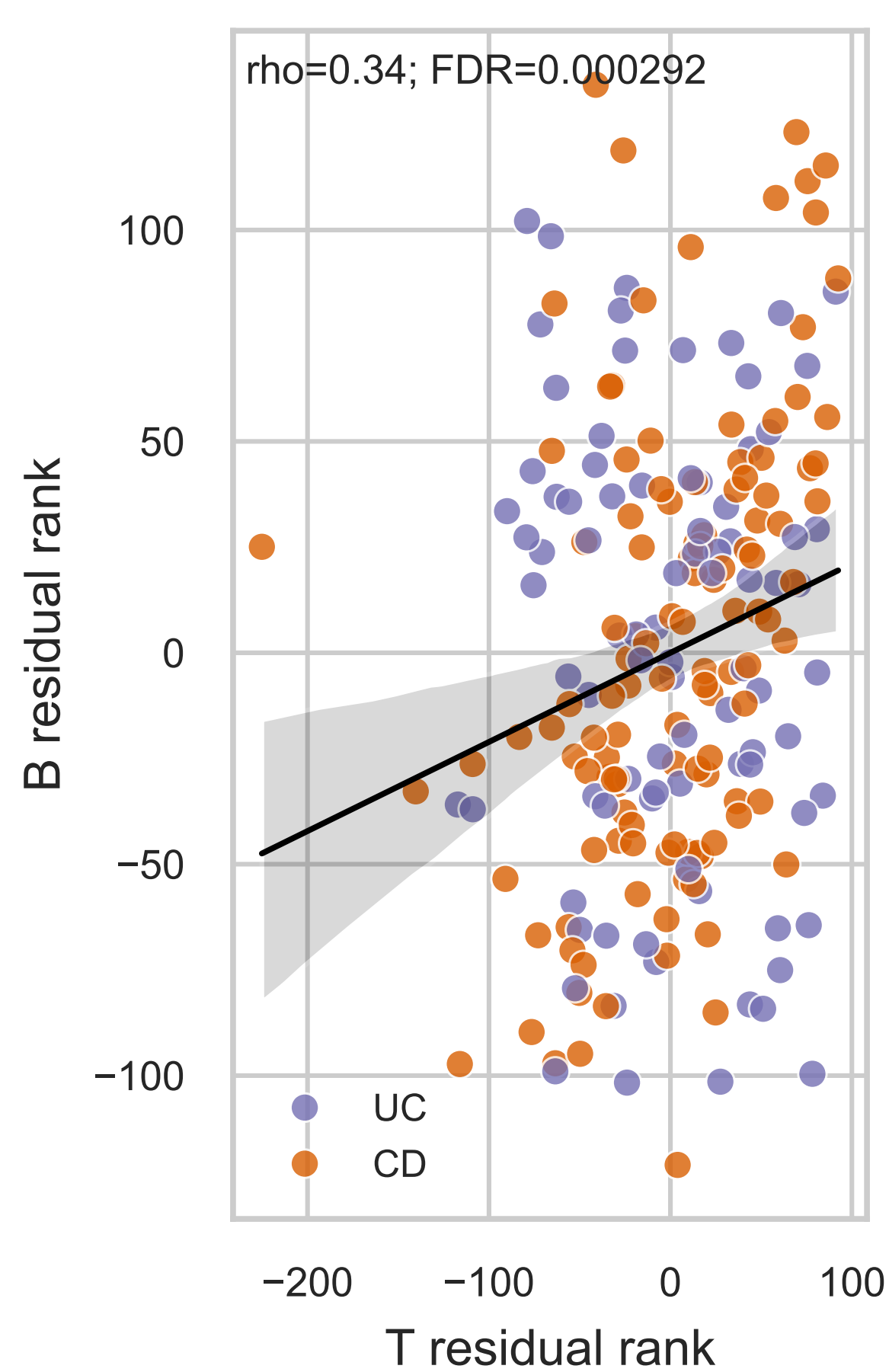

bioRxiv preprint doi: https://doi.org/10.64898/2026.09.20.753043; this version posted September 23, 2026. The copyright holder has placed this preprint (which was not certified by peer review) in the Public Domain. It is no longer restricted by copyright. Anyone can legally share, reuse, remix, or adapt this material for any purpose without crediting the original authors.

H Acquisition-series robustness

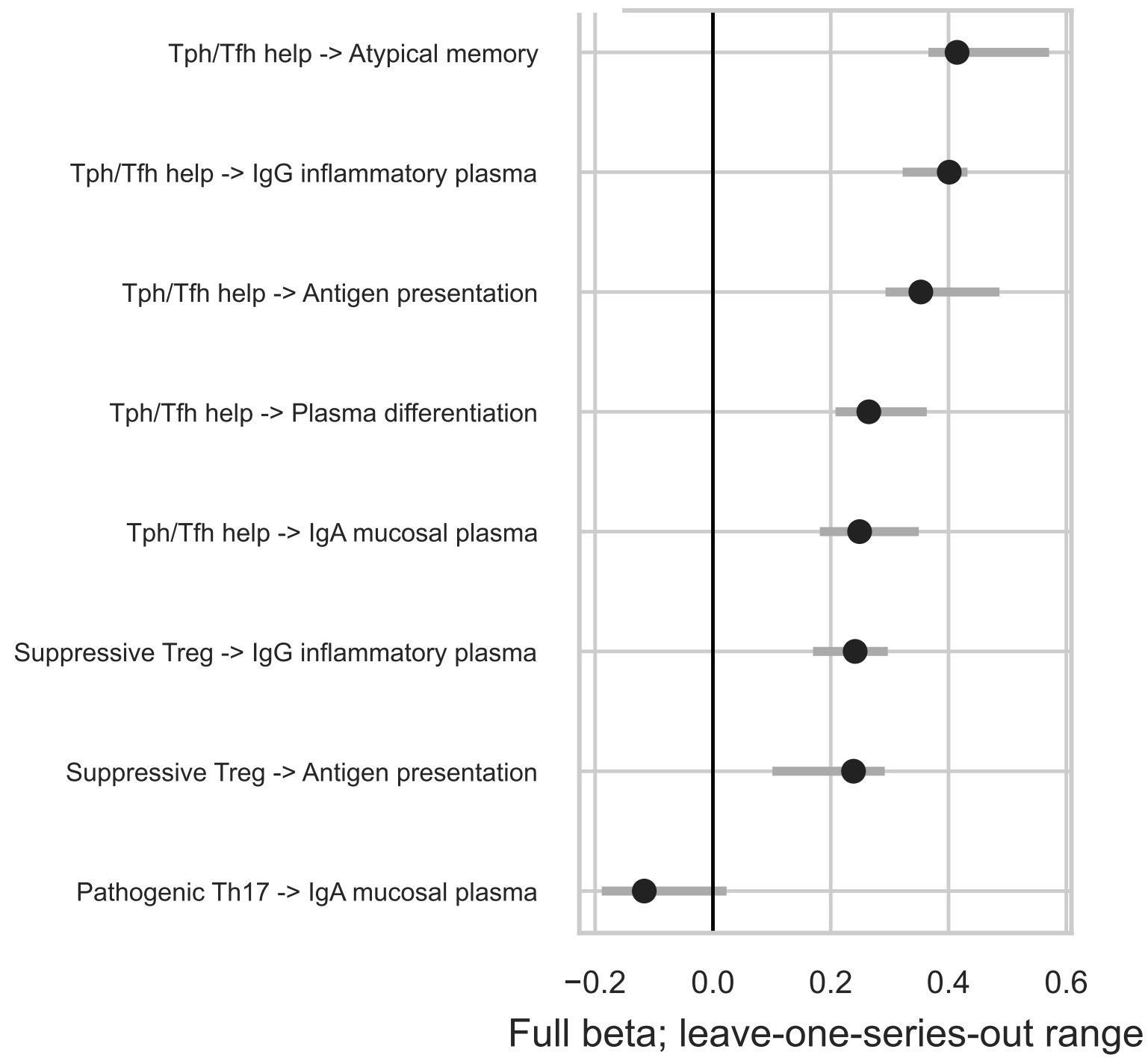

I Helper-B-cell interaction estimates across shared acquisition series

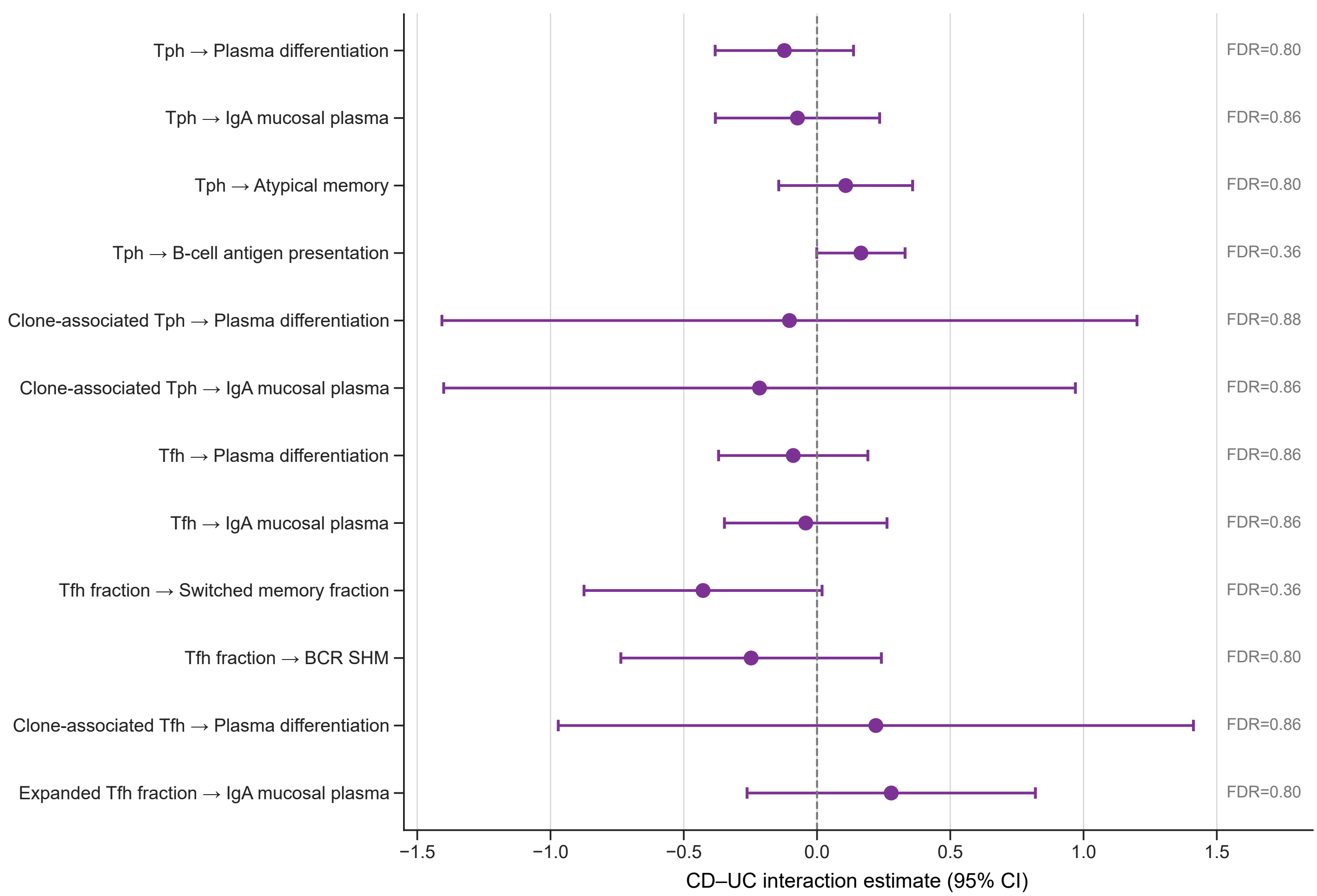

bioRxiv preprint doi: https://doi.org/10.64898/2026.09.20.753043; this version posted September 23, 2026. The copyright holder has placed this preprint (which was not certified by peer review) in the Public Domain. It is no longer restricted by copyright. Anyone can legally share, reuse, remix, or adapt this material for any purpose without crediting the original authors.

Figure S9. External mucosal and cross-tissue validation

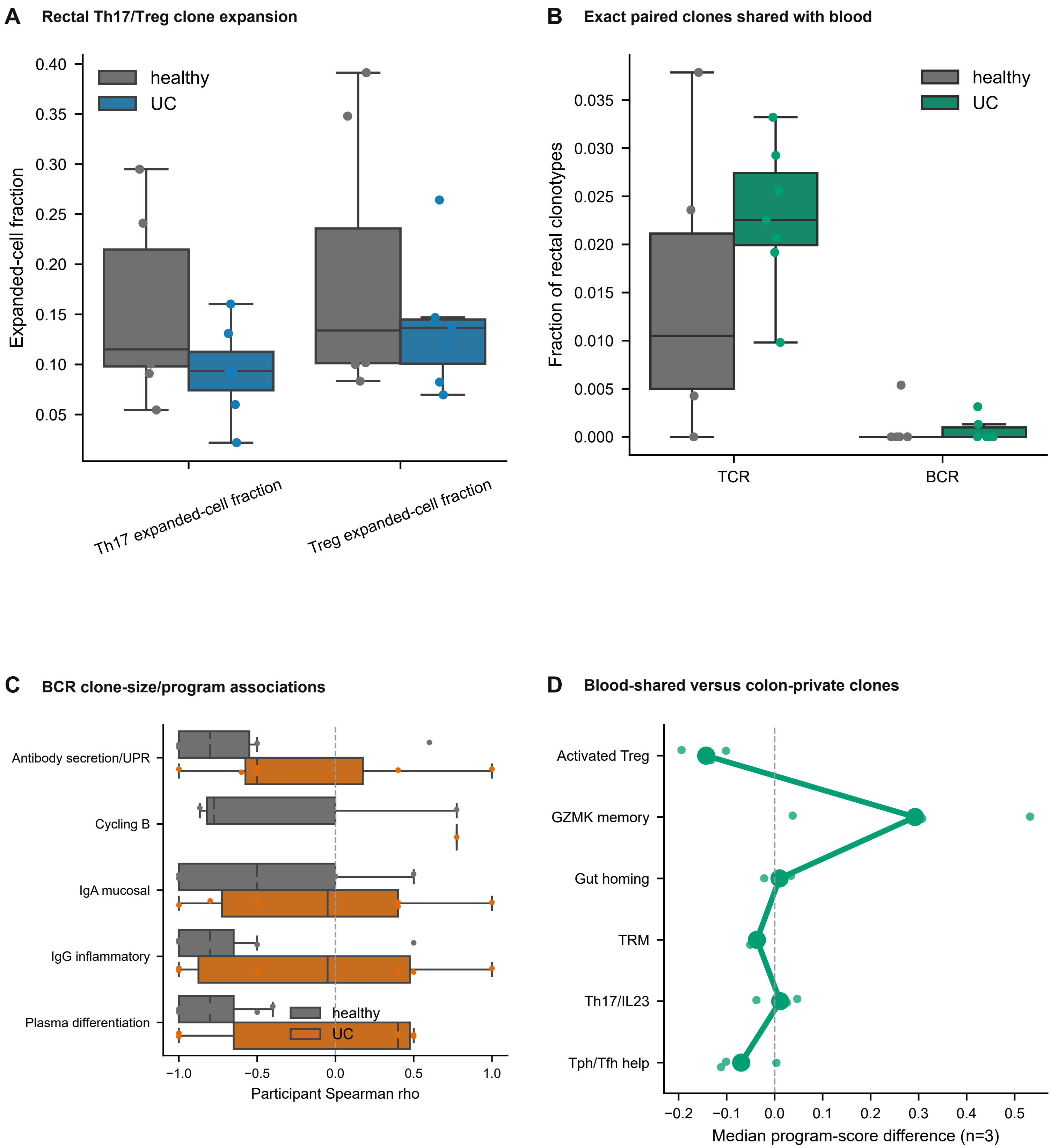

bioRxiv preprint doi: https://doi.org/10.64898/2026.09.20.753043; this version posted September 23, 2026. The copyright holder has placed this preprint (which was not certified by peer review) in the Public Domain. It is no longer restricted by copyright. Anyone can legally share, reuse, remix, or adapt this material for any purpose without crediting the original authors.

Figure S10. Nested machine-learning validation

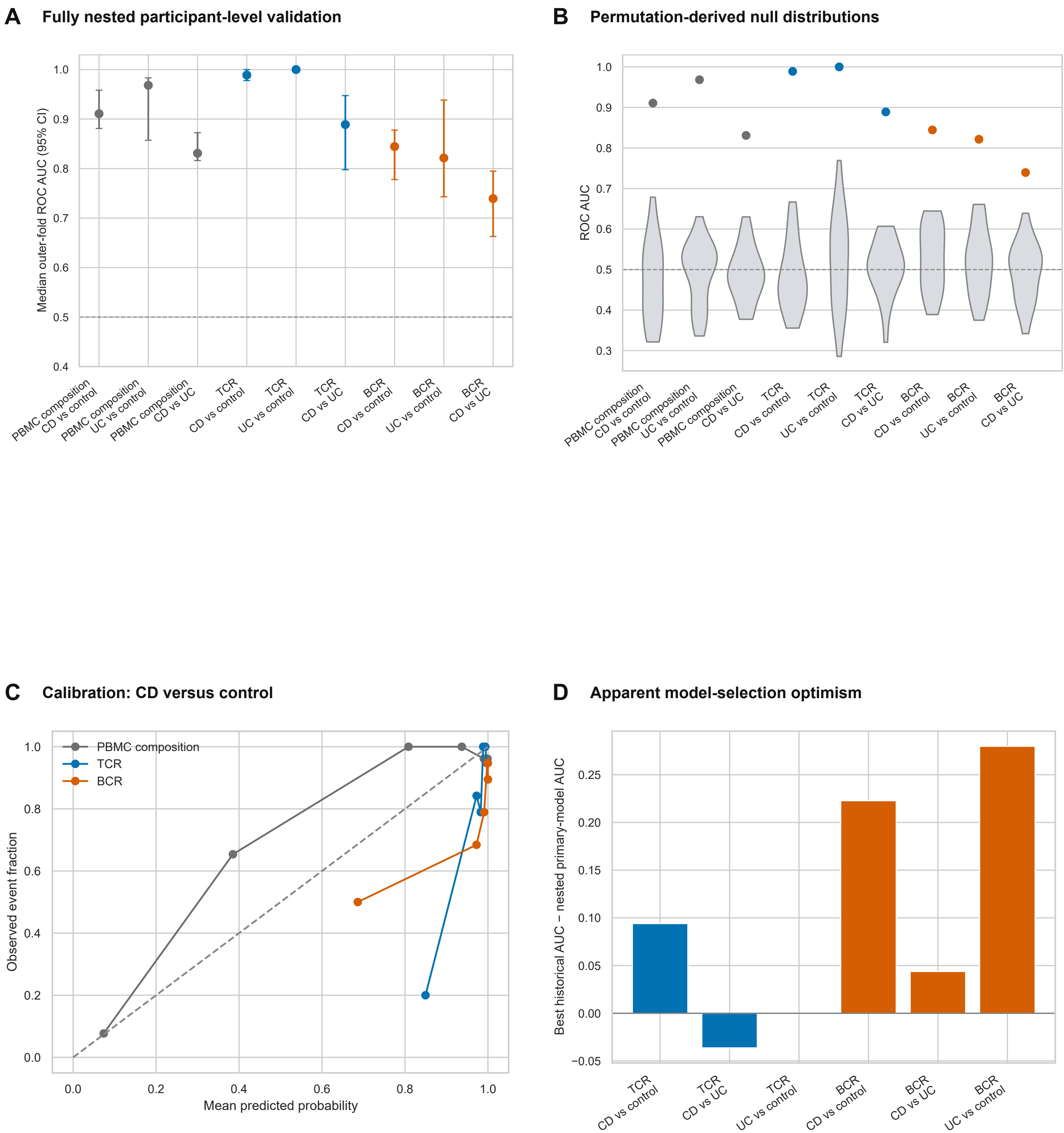

Supplement: Supplementary file 1 [file NIHPP2026.09.20.753043v1-supplement-1.pdf]
